# Detour to success: photoswitching *via* indirect excitation

**DOI:** 10.1039/d4sc02538e

**Published:** 2024-07-02

**Authors:** Kim Kuntze, Jussi Isokuortti, Jacob J. van der Wal, Timo Laaksonen, Stefano Crespi, Nikita A. Durandin, Arri Priimagi

**Affiliations:** a Faculty of Engineering and Natural Sciences, Tampere University Tampere Finland arri.priimagi@tuni.fi nikita.durandin@tuni.fi; b Department of Chemistry, University of Texas at Austin Austin TX USA; c Department of Chemistry, Ångström Laboratory, Uppsala University Uppsala Sweden stefano.crespi@kemi.uu.se; d Faculty of Pharmacy, University of Helsinki Helsinki Finland

## Abstract

Photoswitchable molecules that undergo nanoscopic changes upon photoisomerisation can be harnessed to control macroscopic properties such as colour, solubility, shape, and motion of the systems they are incorporated into. These molecules find applications in various fields of chemistry, physics, biology, and materials science. Until recently, research efforts have focused on the design of efficient photoswitches responsive to low-energy (red or near-infrared) irradiation, which however may compromise other molecular properties such as thermal stability and robustness. Indirect isomerisation methods enable photoisomerisation with low-energy photons without altering the photoswitch core, and also open up new avenues in controlling the thermal switching mechanism. In this perspective, we present the state of the art of five indirect excitation methods: two-photon excitation, triplet sensitisation, photon upconversion, photoinduced electron transfer, and indirect thermal methods. Each impacts our understanding of the fundamental physicochemical properties of photochemical switches, and offers unique application prospects in biomedical technologies and beyond.

## Abstract 2

Photoswitching *via* indirect excitation allows harnessing low-energy photons to control molecular configuration. Herein, the state of the art of the predominant indirect photoswitching methods is presented.

## Graphical abstract

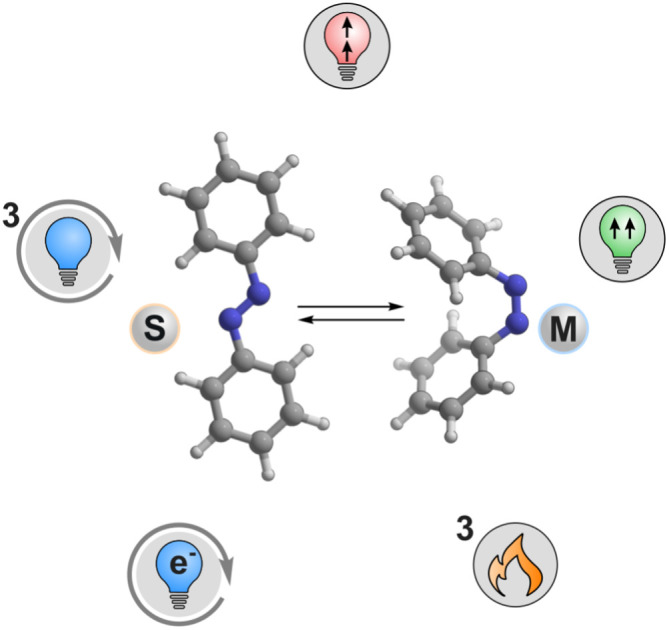

## Introduction

**Fig. 1:**
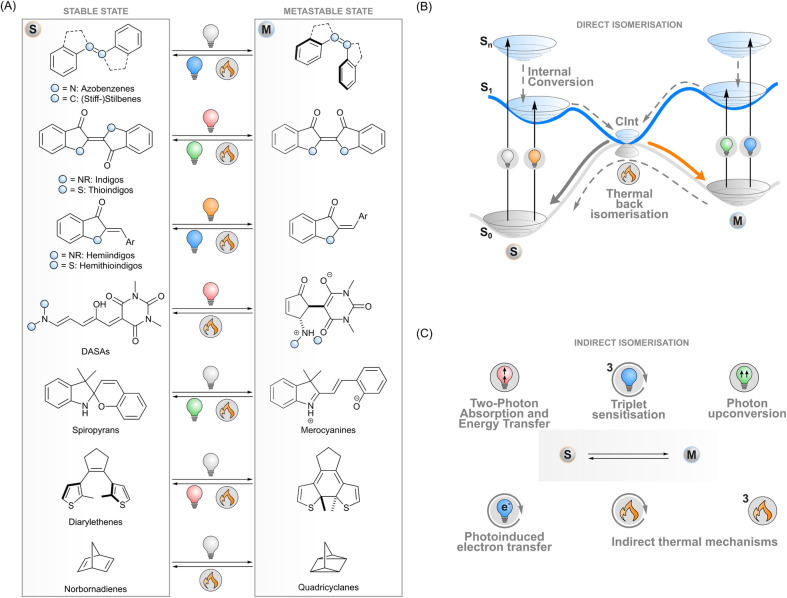
(A) Selected photoswitch families, showing the stable and metastable isomers as well as typical stimuli used to bring about the isomerisation. (B) A schematic view of the processes related to isomerisation with direct excitation. (C) Isomerisation methods utilising indirect excitation, *i.e.* excitation of a proxy system that induces the isomerisation.

Photoswitches are molecules that convert reversibly between isomers upon excitation with light. A key example of a naturally occurring photoisomerisable molecule is retinal that undergoes *Z* → *E* isomerisation after absorbing a photon, which initiates the cellular signalling cascade responsible for vision.^1^ During the last century, chemists have designed a myriad of artificial photoswitch structures: azobenzenes,^2^ (stiff-)stilbenes,^3^ indigoids,^4^ diarylethenes,^5^ norbornadienes/quadricyclanes,^6^ spiropyrans/merocyanines,^7^ and donor–acceptor Stenhouse adducts (DASAs),^8^ to name a few (Fig. 1A). The physicochemical properties of a switch change upon isomerisation and give rise to photoresponsive functions. For instance, the *E*–*Z* isomerisation of azobenzenes, stilbenes and indigoids can be utilised to control the supramolecular interactions of molecular systems or to induce strain into a macroscopic material. The electrocyclisation of diarylethenes and spiropyrans, on the other hand, drastically changes the conjugation and dipole moment of these molecules, respectively. These phenomena can be exploited in photoresponsive systems operating on the molecular or macroscopic level in medicine,^9^ biosciences,^10,11^ catalysis,^12^ energy storage,^13^ electronics,^14^ optics,^15^ 3D printing,^16^ soft robotics^17^ and other materials applications.

In general terms, photoisomerisation is based on exciting a molecule to an excited singlet state by irradiating one of its absorption bands. Subsequent events on the potential energy surfaces then lead to isomerisation with a certain efficiency *i.e.* isomerisation quantum yield (Fig. 1B). If the isomers have distinct absorption profiles, it is possible to favour isomerisation in one direction by choosing an appropriate excitation wavelength, leading to photostationary states where one of the isomers is enriched. One isomer typically has a higher ground-state energy and is denoted as the metastable isomer. Thermal back-isomerisation to the stable isomer is typically observed as well; ground-state isomerisation half-lives range from nanoseconds to years and thus partly determine the research directions to which each photoswitch is applicable.^5,16^

The majority of photoswitches suffer from one critical limitation which hinders their applicability on different research fronts: at least one of the two isomers absorbs only ultraviolet light. This high-energy irradiation often suffers from unwanted absorption or scattering by the matrix surrounding the photoswitches, which causes limited penetration depth and spatial resolution, may have a degrading effect on many materials, and is particularly detrimental to living systems. The ability to control the isomerisation with visible light or with other stimuli would thus be beneficial for a range of applications.^18–20^ In medicinal and biotechnological applications, the excitation wavelengths should optimally fall inside the bio-optical window located in the red and near-infrared light region (>650 nm) where penetration depths through tissue are the best. To meet these requirements, the absorption bands of conventional photoswitches such as azobenzenes can be red-shifted through structural modifications,^18^ albeit often at the expense of reduced thermal stability of the metastable isomer,^21,22^ which limits the scope of these switches. Novel photoswitch scaffolds that inherently absorb red light such as DASAs,^23^*N*,*N*-difunctionalised indigos^24^ and *peri*-anthracenethioindigos^25^ have also been designed, but so far, they have not reached the versatility of more conventional photoswitches in terms of applications.

To overcome these limitations of directly exciting the photoswitch core, another approach is possible. Indirect isomerisation leaves the intrinsic properties (such as the stability of the metastable state) of the photoswitch intact but expands the excitation modality beyond its capabilities by introducing a proxy system responsible for harvesting the excitation energy and transferring it to the photoswitch (Fig. 1C). When designed properly, this enables photoisomerisation with lower-energy photons than absorbed by the photoswitch. Several concepts for indirect excitation of photoswitches have been demonstrated: two-photon excitation of an antenna moiety and subsequent Förster (singlet) energy transfer to the photoswitch,^26,27^ triplet sensitisation,^28,29^ photon upconversion,^30,31^ and photoinduced electron transfer.^32^ These photochemical pathways are often accompanied by thermal mechanisms that may have undesired effects on the switching system but could also provide an additional way of tuning the isomerisation dynamics. To fully harvest the potential of these strategies, a deep understanding of the underlying mechanisms is paramount.

In this perspective we shall explore these indirect photoisomerisation strategies from theoretical and practical points of view, giving insight into their fundamental mechanisms and demonstrating how they have been exploited to enable photoswitching with low-energy light. We will cover various photoswitch families with a particular focus on azoarenes and stilbenes and guide the reader to recent reviews regarding some other photoswitch families such as diarylethenes and norbornadienes.^33,34^ We also delve into the often-overlooked thermal isomerisation routes and propose how they can be either suppressed or exploited. Finally, we provide an outlook on the remaining challenges and opportunities in this promising field of research.

### Indirect photoexcitation mechanisms

All the indirect photoexcitation mechanisms described hereafter involve a photon absorber (a sensitiser) that may be a molecular fraction covalently linked to the photoswitch or an independent molecule (or nanoparticle). To understand the role of the sensitisers in indirect photoswitching, it is important to first understand the energy transfer pathways involved. After initial excitation to the first excited singlet state (^1^Sens*), the sensitiser can relax back to the ground state thermally or radiatively (internal conversion or fluorescence, respectively), or it can undergo intersystem crossing (ISC) to the lower-energy first excited triplet state (^3^Sens*). Since ISC involves a change in the spin state of the sensitiser, it can only compete with the singlet → singlet transitions when the spin–orbit coupling is strong. The most common examples of dyes that exhibit efficient ISC are porphyrin and phthalocyanine complexes that contain transition metals such as palladium or platinum. If crossing to the triplet state occurs, the excited state lifetime is typically prolonged by orders of magnitude due to the low probability of the spin-forbidden ^3^Sens* → ^1^Sens transition.

Out of the four indirect excitation mechanisms described below, triplet energy transfer and photon upconversion depend on the sensitiser's triplet state, whereas two-photon absorption and subsequent energy transfer is typically a singlet process. Electron transfer reactions are possible with both spin states, although the pathway is more efficient if the longer-living triplet state can be formed, especially in diffusion-controlled systems. In the next sections, we will cover these indirect isomerisation approaches separately, first going through the photochemistry of the sensitiser and then following up with the isomerisation mechanism of the switch.

### Two-photon absorption and subsequent energy transfer

**Fig. 2:**
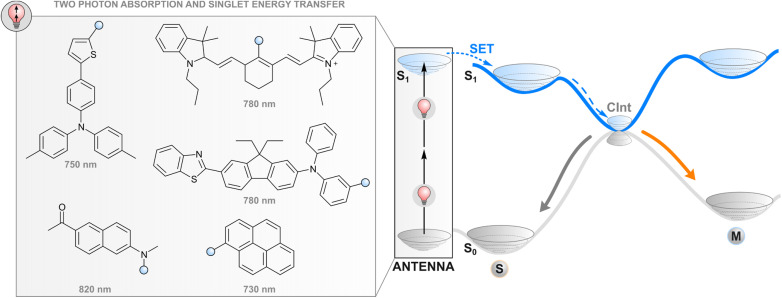
Two-photon excitation of a covalently linked absorber (antenna), subsequent singlet energy transfer to the photoswitch, and the following events in the excited state.

One way to avoid the use of high-energy photons for photoswitching is two-photon absorption where two light quanta, each with half the energy required for one-photon excitation, are absorbed (quasi-)simultaneously. While direct two-photon excitation of a photoswitch is in principle possible for any structure and has been used for 3D data storage^35^ and surface microstructuring,^36^ pulsed light with extremely high peak intensity (even TW cm^−2^) is typically required due to low nonlinear absorption cross-sections. Lower intensities may be applied for some switches with push–pull motifs that increase the probability of simultaneous absorption.^37^ More generally, two-photon excitation of a photoswitch can be achieved indirectly (Fig. 2) by utilising a molecular fragment with a high nonlinear absorption cross-section, and the capability to act as a donor in resonance energy transfer to the photoswitch, *i.e.* a high fluorescence quantum yield, favourable relative orientation between the donor and photoswitch transition dipole moments, and emission that overlaps with the absorption spectrum of a photoswitch. Commonly, large π systems such as naphthalene, pyrene, or anthracene derivatives are utilised for this purpose, often with a donor–acceptor nature that increases the nonlinear absorption cross-section.^38^ After two-photon excitation with low-energy light, the singlet-excited absorber transfers its energy to the photoswitch, typically through the aforementioned Förster resonance energy transfer, leading to a singlet-excited photoswitch molecule. Subsequent events on the excited state potential energy surface of the photoswitch lead to isomerisation. The resulting photostationary state is determined by the overlap between the emission spectrum of the two-photon absorber and the absorption spectra of the photoswitch isomers, as well as the quantum yields of relaxation to either isomer from the excited state.

Efficient photoswitching based on indirect two-photon excitation, typically with wavelengths from 750 to 800 nm, has been demonstrated for azobenzenes,^39,40^ azoheteroarenes,^41^ overcrowded alkene motors,^42^ and diarylethenes,^26^ highlighting the universal applicability of the method. Potential applications have also been shown. Utilising azobenzene nanoimpellers that physically trap anticancer agents inside mesoporous SiO_2_ nanoparticles as the *E* isomer, *E* → *Z* isomerisation *via* indirect two-photon excitation led to *in vitro* cancer cell death with 760 nm light irradiation.^27^ Also activation of light-gated ion receptors has been accomplished in living cells *via* indirect two-photon excitation, enabling neuronal and astrocytic stimulation.^43^ Also a photochromic imidazole dimer has been isomerised with two-photon excitation,^44^ but in that case the absorption of photons occurs sequentially. The mechanism furthermore proceeds through charge transfer and is hence highlighted in the section covering photoinduced electron transfer.

Since the process does not involve crossing to a triplet state, the difficulties related to the quenching of the excitation by molecular oxygen are avoided (*vide infra*). On the other hand, efficient energy transfer typically requires covalent linkage to the photoswitch core due to the short lifetime of the excited singlet state of the absorber as compared to triplet sensitisers. Although extreme excitation intensities can be avoided by utilising state-of-the-art nonlinear absorbers, the required intensities are still orders of magnitude larger than those needed by other indirect excitation methods or direct one-photon excitation, and require focused laser beams.^37^ For this reason, the utility of two-photon processes is limited in, for instance, photoresponsive materials that would require fast and efficient photoswitching simultaneously on a large area. However, the need for a focused photon flux can also be seen as an asset, as it enables a very high (sub-μm-scale) spatial precision in photoisomerisation, which is advantageous for micro- and nanolithography, some drug delivery systems and the manipulation of intra- and extracellular processes in specific parts of the tissue.^37^

### Triplet sensitisation

**Fig. 3:**
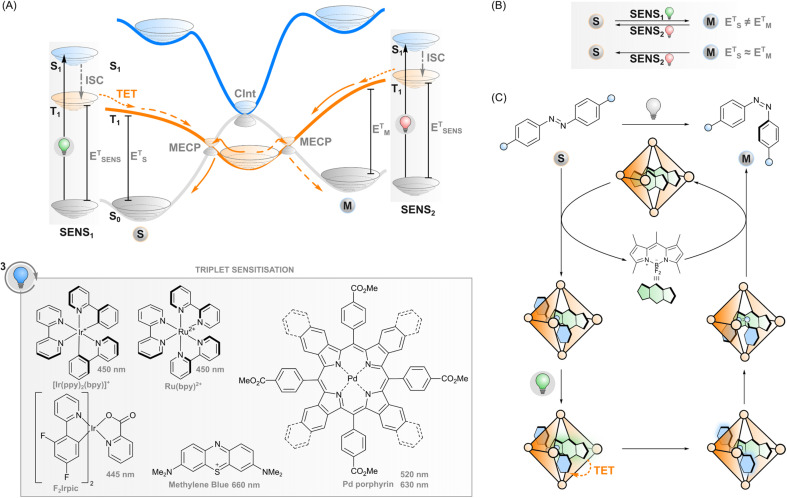
(A) Triplet sensitisation: excitation of the sensitiser (SENS), intersystem crossing (ISC) to its triplet state, triplet energy transfer (TET) to the photoswitch, and ultimately relaxation to the ground state *via* one of the minimum energy crossing points (MECPs). Selected sensitisers and their absorption maxima are presented. (B) If the triplet energies of the stable and metastable state (E_S_^T^, E_M_^T^) differ, one of them can be selectively sensitised by choosing a sensitiser with matching triplet energy (E_SENS_^T^), leading to isomerisation from stable to metastable (dashed orange line in (A)) or metastable to stable (solid orange line in (A)). If E_S_^T^ ≈ E_M_^T^, sensitisation leads to the same isomer composition regardless of E_SENS_^T^. (C) DESC utilises flexible coordination cages that can accommodate an *E*-azoarene and a sensitiser but not a *Z*-azoarene. Although the quantum yield of *Z*-azoarene through sensitisation is low, the formed *Z*-azoarenes are expelled from the cage; thus, over time the sensitisation leads to *E* → *Z* isomerisation.

If the excited sensitiser can cross to its triplet state, a new phenomenon may take place: triplet energy transfer (TET) from the triplet-excited sensitiser to a ground-state photoswitch (Fig. 3A), resulting in a singlet ground-state sensitiser and a triplet-excited photoswitch that would be difficult to obtain through direct excitation due to the forbidden nature of the singlet-to-triplet transition. In the case of triplet sensitisation, this transition is allowed since the reaction proceeds *via* electron exchange energy transfer where the total spin is conserved and hence, the total spin multiplicity does not change. Since the Dexter mechanism relies on orbital overlap, the sensitiser and photoswitch need to be in close proximity for the energy transfer to take place. This can be accomplished by covalently linking the two together (which enables mechanisms such as superexchange and hopping^45,46^) or, more commonly, simply relying on collisions of separate molecules, owing to the relatively long lifetime of the triplet state. In this case, the rate of TET depends on diffusion and the electronic coupling between the two molecules. The latter is typically evaluated thermodynamically by comparing the triplet energies of the sensitiser and the photoswitch: larger exoergic gap Δ*E* from the sensitiser to the photoswitch leads to a higher rate of triplet energy transfer. Traditionally, this has led to the design principle of triplet photosensitised systems where the triplet energy of the sensitiser should be higher than that of the acceptor/photoswitch. However, we will explore the limits of this energetic requirement in this perspective.

After sensitisation to the triplet-excited state, the events depend on the potential energy surfaces of the photoswitch (Fig. 3A) and, subsequently, the rates of intersystem crossing to the ground state of either isomer. In the case of azobenzenes, the T_1_ state is barrierless and crosses S_0_ at two N=N twist angles, approximately 65° and 120°, with the T_1_ minimum (at 110°) close to the latter. Furthermore, the crossing point at 120° and the T_1_ minimum are nearly degenerate, while a small energy barrier exists between the 65° crossing and T_1_ minimum.^47,48^ This clearly indicates that isomerisation *via* excitation of the T_1_ strongly favours the *E* isomer.^28,49,50^ As the product of the almost negligible energy barrier and considerable spin–orbit coupling, overall T_1_–S_0_ intersystem crossing in azobenzene is very rapid with an estimated rate constant of 10^11^ s^−1^.^47^ Against this backdrop, the triplet sensitisation of azobenzene offers an efficient mode for rapid and selective *Z*–*E* isomerisation, yielding a PSS with >95% *E*. Triplet sensitisation is relatively universal as the triplet energy of azobenzenes is relatively unperturbed by structural modifications.^50^ However, azoheteroarenes such as arylazopyrazoles^51^ and the *Z* isomer of diazocines (bridged azobenzenes)^52^ exhibit considerably higher triplet energies than conventional azobenzene or the corresponding *E* isomers. This may be an obstacle to using low-energy light but also an asset when selective sensitisation of either isomer is desired.

Although the phenomenon has been known for decades, only recently have triplet sensitisers such as methylene blue^32^ and transition metal porphyrin and phthalocyanine complexes^29,53^ been used to enable red light excitation (*via* exciting at their Q-bands). The higher-energy S_0_-to-S_2_ transition (also known as the Soret band) of the porphyrin photosensitiser in the blue end of the visible spectrum may overlap with the absorption of the azobenzene and cause incomplete *E* → *Z* isomerisation. However, by judicious selection of the photoswitch/sensitiser pair with minimised spectral overlap and by tuning their concentration ratios, systems that use visible light for both direct *E* → *Z* (525 nm) and triplet-sensitised *Z* → *E* isomerisation (770 nm) can be realised.^29^ Although the triplet energies of porphyrins that absorb red and near-infrared light are rather low and in some cases even lower than that of the photoswitch, sensitisation can be efficient due to entropic contributions and negligible back-energy transfer from the azobenzene, owing to its ultrashort triplet lifetime, to the sensitiser. In addition to different azobenzenes (including *ortho*-fluorinated ones), also the *Z* → *E* isomerisation of arylazopyrazoles was recently induced with red light (630 nm), using a palladium porphyrin sensitiser.^51^

Furthermore, in an important step forward from purely fundamental solution studies, the use of a red-light-absorbing porphyrin or a triplet-energy-deficient phthalocyanine in TET-catalysed azobenzene isomerisation was recently demonstrated in a bioplastic material.^53^

Besides azobenzenes, only one other type of photoswitch has seen such extensive characterisation in the triplet manifold. Stilbene has been the subject of triplet sensitisation studies since the 1960s.^54,55^ The triplet-sensitised isomerisation of olefins in general is an important photochemical transformation in alkene synthesis, and we would like to point interested readers to the recent (2022) review.^56^ Unlike azobenzene, stilbene undergoes triplet-sensitised isomerisation readily in both directions (Fig. 3B). The triplet state energies of both isomers are considerably higher than in azobenzene, 2.14 and 2.35 eV for *E* and *Z* isomers, respectively.^54^ Thus, relatively selective isomerisation can be achieved by selecting a sensitiser with triplet energy close to the desired acceptor isomer. Sensitisation thus leads to a mixture of isomers whose isomer composition depends on the relative triplet energy of the sensitiser to both isomers.

Delightfully, triplet sensitisation of stilbene isomerisation has recently leapt from fundamental research to applications. Photoactuation of a polymer film doped with an iridium triplet sensitiser where the stilbene moiety was part of the polymer chain was demonstrated.^57^ Most importantly, this approach afforded a substantial redshift from UV (365 nm) to visible blue (445 nm) in the excitation wavelength. In photopharmacology, a ruthenium photosensitiser was used to isomerise several stilbene-based drug molecules to achieve selective cytotoxicity and disruption of the cellular microtubule network under 450 nm (one-photon) or 916 nm (two-photon) excitation of the sensitiser.^58^ So far, these works serve as the crucial first steps towards applications of triplet-sensitised photoswitching, more of which are surely to follow, due to the apparent milder, redshifted illumination conditions. In the future, the inherently high spatial resolution of triplet sensitisation (in the order of nanometer) could be of fundamental interest in looking at, *e.g.*, intracellular processes or pathways with a confined triplet sensitiser.

In addition to azobenzene and stilbene, several other types of *E*–*Z* photoswitches have been shown to undergo isomerisation *via* triplet sensitisation. In general, triplet-sensitised isomerisation can be expected for molecules that can rotate about a C=C, N=N or C=N^59^ bond. As such, triplet-sensitised isomerisation has been demonstrated for overcrowded-alkene-based molecular motors,^60^ hydrazones^61^ and thioindigos.^62^ Compared to *E*–*Z* isomerisation, photoswitches that undergo electrocyclisation are underrepresented in the triplet manifold. Triplet-sensitised isomerisation of norbornadienes/quadricyclanes and diarylethenes were recently reviewed,^33,34^ and we gladly point the interested reader their way. In addition to these two types of electrocyclisation photoswitches, also spiropyrans/merocyanines have been demonstrated to be capable of isomerisation upon triplet energy transfer.^63^

Regarding azoarenes, we would like to highlight two special cases of triplet isomerisation: bridged azobenzenes (also known as diazocines) and DESC (disequilibration of azoarenes under confinement). A bridged azobenzene, unlike a regular azobenzene, has substantially different triplet energies in *E* (1.53 eV) and *Z* (1.89 eV) isomers. This property enables, like in the case of stilbene, selective sensitisation of either isomer, which has recently been utilised to construct a purely triplet-sensitised bidirectional photoswitching system, *i.e.* without direct excitation of the photoswitch. PSS isomer distributions of 49% *E* and >99% *Z* could be acquired upon excitation with 530 and 740 nm, respectively.^52^ The other special case of triplet-sensitised photoswitching leads to the metastable isomer exclusively, a previously unattained feat. The DESC approach (Fig. 3C) utilises confinement to drive the isomerisation of virtually any azoarene from *E* to *Z* – even in aqueous environments. Flexible coordination cages are used to confine the *E*-azoarene and a triplet sensitiser. Following excitation of the sensitiser with visible (up to 635 nm) light, intersystem crossing and TET to the azoarene, the photoswitch can either relax back to the *E* isomer or the *Z* isomer. The confinement may induce some pre-twisting of the azoarene, which increases the quantum yield of the latter possibility, but it is still rather low. However, after isomerisation to the bent *Z* isomer, the cage cannot accommodate the azoarene anymore, and it is expelled from confinement. Over time, the system will thus efficiently isomerise the switches from *E* to *Z*, in many cases more efficiently than *via* direct excitation.^64^

The power of triplet sensitisation lies in its universality: it can be used to isomerise several photoswitch families, and since inside a given photoswitch family (*e.g.*, azobenzenes) the triplet energy is apparently little affected by structural changes, the same sensitiser can be used for almost any compound. The biggest drawback in indirect isomerisation *via* TET is its susceptibility to quenching by molecular oxygen which exists as a triplet in its ground state and is readily excited to the singlet state by visible-light triplet sensitisers,^65^ which in turn hampers the sensitisation of the photoswitch molecules. In addition, singlet oxygen is extremely reactive and may degrade either the azobenzene or the sensitiser. As the concentration of oxygen in ambient solutions lies in the range of millimoles per litre, any experiments featuring micromolar concentrations of the sensitiser–azobenzene system are seriously affected without precautions. Common measures to tackle the problem include the deaeration of the solvent *via* freeze–pump–thaw cycles or sparging with an inert gas, and the use of oxygen scavengers.^29^ In the aforementioned solid-state study, the sensitiser and azobenzene molecules were confined within a gelatine matrix that blocks the diffusion of oxygen, thus removing the need for further deaeration steps.^53^ Similarly, the confinement in DESC hinders the diffusion of oxygen to the sensitiser–azoarene pair and at the same time ensures the proximity of the triplet energy donor and acceptor. This allows for a fast (on the ns timescale) TET process that successfully competes with oxygen quenching.^64^

Although the triplet sensitisation of switches like azobenzenes, stilbenes and norbornadienes has been thoroughly studied, several new discoveries have been made in the last few years, indicating that much progress is still to come. Systematic fundamental studies are needed for these well-known molecular families and especially for less explored switches to determine how electronic and conformational changes modify their triplet properties and whether the insight gained into the excited state properties of conventional switches can be generalised for other families. Furthermore, more applied research lines should be ventured to bring the fundamental research into practice. For example, utilising longer wavelengths will be undoubtedly beneficial for biomedical applications, *e.g.* light-triggered drug release^66^ and imaging^67^ or in chemical biology as a sensory tool.^68^ However, caution must be taken since both at *in vivo* and *in vitro* conditions the concentration of oxygen is sufficient to quench the triplet state, leading to the formation of reactive oxygen species. This in turn can result in either degradation of the photoswitch and/or the sensitiser or even the system under study. Therefore, we foresee that this approach will be utilised for, *e.g.*, energy storage and release devices, sensors, and biomedicine applications for which hypoxic/anoxic conditions are relevant. Intriguingly, inherent oxygen sensitivity could be turned into an advantage by using triplet-based photoswitching to target hypoxic disease sites, *e.g.* solid tumours, which poorly respond to conventional photodynamic therapy.

### Photon upconversion

**Fig. 4:**
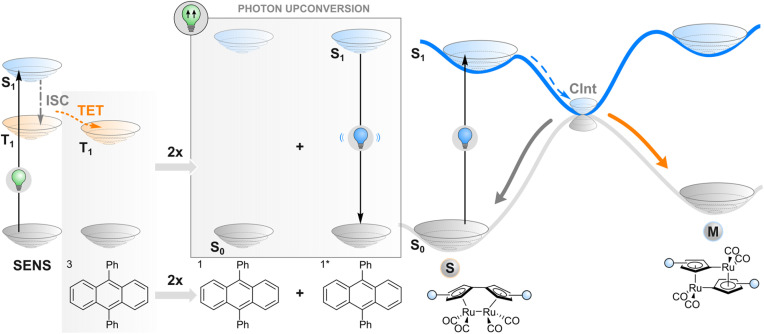
Triplet–triplet annihilation upconversion. Following the excitation of a sensitiser (SENS) and triplet energy transfer (TET) to an annihilator, two triplet-excited (T_1_) annihilators react to form one singlet-excited (S_1_) annihilator. It fluoresces light that is absorbed by the photoswitch, which ultimately leads to isomerisation from stable to metastable state.

As an alternative to TET described in the previous section, one may employ a more complex phenomenon also dependent on a triplet state: triplet–triplet annihilation upconversion (TTAUC) or triplet fusion upconversion.^69^ The beauty of this process lies in the fact that it allows the generation of high-energy photons (*e.g.*, blue) upon excitation of a system with low-energy photons (*e.g.*, red light) with a theoretical quantum yield of 50%. To make it happen, two types of molecules (in addition to the photoswitch) are usually needed, a sensitiser and an annihilator. Upon excitation of a sensitiser molecule, it undergoes intersystem crossing to generate a triplet-excited state and subsequent TET to an annihilator molecule. Then two triplet-excited annihilator molecules may collide, leading to triplet fusion (annihilation) where one singlet excited state and one singlet ground state of annihilator will be formed. The singlet excited state of an annihilator will eventually emit a photon of higher energy than initially absorbed by the sensitiser, resulting in anti-Stokes emission that can be absorbed by the switch (Fig. 4). Thanks to a wide range of TTAUC sensitiser/annihilator pairs including commercially available ones,^69–71^ excitation and emission wavelengths can be tuned to induce the isomerisation of a photoswitch of interest. In contrast to two-photon absorption, the process of TTAUC is efficient even by using non-coherent, low-intensity light, including the sun.^72^ A potential hurdle to utilising TTAUC for photoswitching is TET from the sensitiser to the photoswitch rather than to the annihilator, leading to triplet sensitisation rather than photon upconversion. This may be avoided by physically separating the TTAUC pair and the photoswitch, or by using upconversion nanoparticles (UCNPs, typically based on NaYF_4_ doped with lanthanides) instead of organic molecules (*vide infra*).

The first example of utilising TTAUC for photoswitching was published in 2013. A sandwich-like microfluidic device was created for solar energy storage consisting of two chips, one of which contained the TTAUC pair and the other the photoswitch, a fulvalene diruthenium derivative. The upconversion system depended on the absorption of 500–550 nm green light by a palladium(ii) porphyrin derivative and consequent upconverted emission of violet light by a diphenylanthracene annihilator, which ultimately transformed the photoswitch into its higher-energy isomer capable of storing the light energy.^30^ Since then, similar approaches (with slightly different TTAUC pairs in different environments) have been utilised to isomerise a diarylethene,^73^ a styryl hemicyanine barium(ii) complex,^74^ an azotolane crosslinker for photoactuation,^31^ and a hemiindigo.^75^ In the latter case, the authors were able to indirectly induce isomerisation in both directions by using a pH-responsive TTAUC system where the wavelength of the upconverted light could be tuned by adding acid or base.

TTAUC has also been combined with other phenomena covered in this perspective. Two different approaches were utilised to induce the isomerisation of stilbene with red (635 nm) light either from *Z* to *E* or from *E* to *Z*. For the former direction, an annihilator was used that, in its singlet excited state, could abstract an electron from the stilbene and thus catalyse the isomerisation to the more stable *E* form (photoinduced electron transfer; *vide infra*).^76^ Contrary to most triplet-fusion-based systems, the TTAUC pair and the photoswitch were not separated physically, which enabled the electron transfer. Isomerisation in the reverse direction was induced by first creating upconverted light in a physically separate vessel and then using this to excite a ruthenium(iii) triplet sensitiser, followed by TET to the photoswitch and finally *E* → *Z* conversion.^77^ A third combination of TTAUC and another photochemical reaction was enabled by tethering a perylene annihilator into a photochromic phenoxyl–imidazolyl radical complex. After the initial excitation and annihilation steps, a fast and efficient intramolecular charge transfer from perylene to the photoswitch takes place, ultimately resulting in the latter's isomerisation after a sophisticated cascade of photochemical processes.^78^

In all the aforementioned TTAUC examples, the TTAUC pair and the photoswitch are either physically separated or the desired photoreaction between the singlet-excited annihilator molecule and the photoswitch is something else than simple emission-reabsorption. However, as already mentioned, the trivial emission of upconverted light without competing photochemical reactions is also possible without physical separation if UCNPs are used instead of TTAUC pairs since the upconversion processes are then confined within the nanoparticles (and often do not even produce triplet states) and do not lead to the triplet sensitisation of the photoswitch molecules. The upconversion inside UCNPs can occur either through a mechanism similar to TTAUC, *e.g.* energy transfer upconversion, or other ones including ground state absorption/excited state absorption, cooperative luminescence, cooperative sensitisation, cross-relaxation, photon avalanche processes, and energy migration-mediated upconversion.^79,80^ Regardless of the mechanism, it results in the emission of upconverted light that can be used to isomerise virtually any photoswitch system *via e.g.* Förster Resonance Energy Transfer (FRET) of simple re-absorption employing singlet manifold of a photoswitch. For more details on UCNP-mediated photoswitching, we would like to direct the reader to a recent (2023) review.^81^ Unfortunately, high power densities (in order of kW cm^−2^) that are needed to reach reasonably high quantum yields (even 1%) hamper the applicability of UCNPs. The excess of excitation energy may lead to, *e.g.*, heating of the sample, which will promote undesired isomerisation of the photoswitch from its metastable isomer to the thermodynamically stable one. However, FRET or re-absorption usually successfully overcompete with the thermal isomerisation effect.^82–84^

### Photoinduced electron transfer

**Fig. 5:**
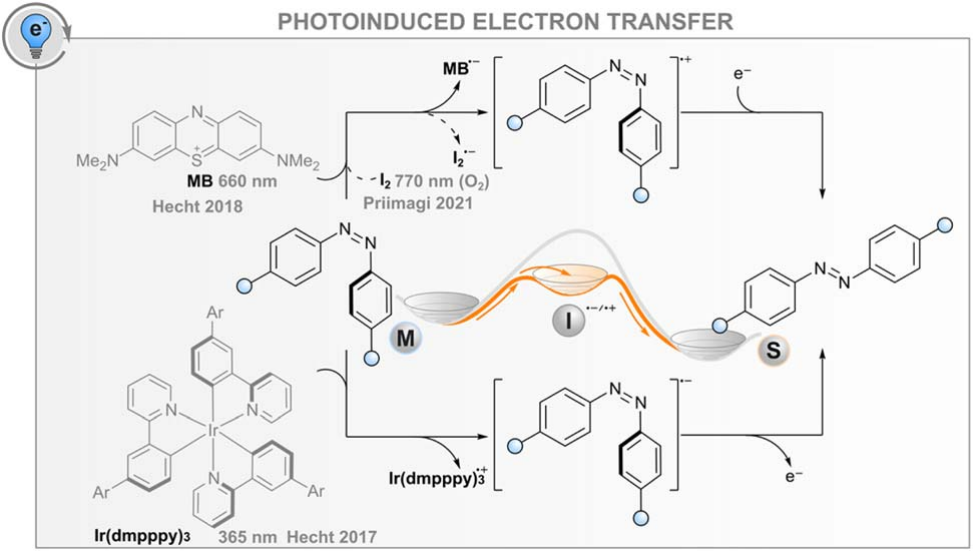
Oxidative (top) and reductive (bottom) photoinduced electron transfer routes.

In addition to the energy transfer processes discussed above, the interaction between an excited photosensitiser and a photoswitch may result in either reductive or oxidative charge transfer (photoinduced electron transfer, PET, Fig. 5); the same effect can also be induced electrochemically without light (*vide infra*). The utility of this process for catalysing the isomerisation is based on the formation of anionic or cationic photoswitch radicals that both isomerise to the thermodynamically more favourable isomer at an extremely fast rate (Fig. 5). In the case of published data for azobenzenes, the ground-state *Z* → *E* isomerisation rates are 10^12^ to 10^17^ times higher than for the respective neutral molecules.^32,85,86^ After isomerisation, the formed cationic or anionic radical *E*-AB˙^(+/−)^ abstracts an electron from another *Z*-azobenzene, thus creating a chain reaction that is evident as isomerisation quantum yields above unity. As a consequence of the high rate of isomerisation and the high quantum yield, the overall reaction rate is governed by diffusion and the efficiency of the initial intermolecular electron transfer step. For the latter, the reduction/oxidation potentials of the excited sensitiser and photoswitch must be sufficiently matched; the oxidative pathway is more probable for electron-rich photoswitches, whereas the reductive one may be useful for electron-poor compounds. The mechanism is promoted by a polar medium that can stabilise the pair of radical ions formed.

Catalysing the *Z* → *E* isomerisation of azobenzenes *via* the reductive route has been demonstrated with an iridium(iii) photosensitiser but only using high-energy excitation (365 nm).^86^ In this regard, an oxidative route seems more feasible: the *Z* → *E* isomerisation of suitably electron-rich azobenzenes can be catalysed with a red-light photosensitiser methylene blue (MB) *via* the formation of an azobenzene radical cation.^32^ For example, 4,4′-dimethoxyazobenzene has an oxidation potential of 1.41 V, matching well the reduction potential of MB in either singlet or triplet excited state (1.56 and 1.60 V, respectively). Thus, MB can be used as a red-light photosensitiser to bring about quantitative and relatively fast isomerisation. It is worth noting that without the sensitiser, complete isomerisation to the *E* isomer is only possible thermally, as direct irradiation leads at best to a PSS with a *Z*-fraction of 31% and even this is only possible with blue light (∼440 nm). Since the system does not depend on triplet states, it tolerates oxygen, albeit with slightly diminished efficiency. As another example, it was shown that the *Z* → *E* isomerisation of moderately electron-rich azobenzenes could be catalysed with molecular iodine with up to near-infrared (770 nm) light.^87^ Surprisingly, the reaction was not only tolerated but mediated by molecular oxygen. The catalytic cycle was proposed to include the formation of singlet oxygen that subsequently abstracts an electron from the azobenzene, initiating the radical cation chain reaction. Iodine seems to have another function, perhaps later in the cycle, since the same oxygen-dependent catalytic reaction was observed when a palladium(ii) phthalocyanine singlet oxygen sensitiser was used instead of iodine, but the reaction was considerably slower.

For both MB and iodine, two major problems exist. The sensitiser absorbs over most of the visible light region, meaning that *E* → *Z* isomerisation of the azobenzene can only be carried out with UV light or the sensitiser must be applied after initial direct photoisomerisation. In addition, hole catalysis is only feasible for suitably electron-rich compounds that match the oxidation potential of the sensitiser (or singlet oxygen): less oxidisable switches do not form the radical cation, whereas too electron-rich compounds over-oxidise irreversibly. Both MB and I_2_ can also catalyse the reaction for compounds without optimal oxidation properties, but in that case, the MB reaction proceeds *via* triplet sensitisation and is impeded by oxygen,^32^ and the I_2_-catalysed reaction relies on the formation of I˙ radicals that are formed when exciting iodine with green light.^88,89^ Additionally, iodine is impractical and not suitable for biological applications due to its toxicity. New photosensitisers should therefore be screened for this purpose.

The aforementioned routes depend, at least partly, on the formation of a relatively long-lived triplet species. The use of exclusively singlet-excited electron transfer agents was only recently studied.^90^ Since the lifetime of a singlet-excited photosensitiser is extremely short, the authors tethered different electron transfer agents to various azobenzenes. They provided the redox potentials and spectroscopic data of 33 azobenzenes and 11 non-phosphorescent (*i.e.*, staying within the singlet manifold) biocompatible chromophores and demonstrated that by combining each azobenzene with a suitable chromophore, it was possible to catalyse the quantitative *Z* → *E* isomerisation of each azobenzene through singlet electron transfer. In some cases, even near-infrared light could be used. Because triplet states were avoided altogether, no quenching by molecular oxygen was observed.

As an example besides the *E*–*Z* isomerisation of double bonds, photoinduced electron transfer has featured in the isomerisation mechanism of a photochromic imidazole dimer.^38^ After sequential two-photon absorption of a porphyrin antenna, electron transfer to the imidazole takes place, ultimately leading to cleavage of the imidazole–imidazole bond. However, examples of purposefully leveraging electron transfer have not been reported widely. Since redox-catalysed isomerisation has already been demonstrated for azobenzenes, stilbenes,^91^ and quadricyclanes,^92^ catalytic conversion from the metastable to stable isomer *via* PET might be feasible for some other photoswitches as well. However, the screening of new photosensitisers with suitable spectral and electrochemical properties is required to broaden the scope of this method to photoswitches of different oxidation potentials and, particularly, to minimise the spectral overlap between the sensitiser and the photoswitch. However, already at its current stage, PET catalysis may enable new opportunities for, *e.g.*, the release of stored solar energy or photoactuation, owing to its ability to induce the back-isomerisation fast, efficiently, and quantitatively, which could be of particular importance in optically dense systems such as polymers, liquid crystals and hydrogels where direct excitation of the whole sample is challenging.

### Thermal mechanisms to control the isomerisation

Until now, we have focused our attention on the population of excited states of photochemical switches by means of an indirect photochemical stimulus that involves another species, *e.g.*, a sensitiser, to drive the isomerisation to completion. Another means of externally controlling the thermal transformation of a switch with a high isomerisation barrier is by altering the potential energy surface that connects the metastable state to the stable state. By doing so, novel thermal pathways involving reaction intermediates can be opened, fine-tuning the thermal lifetime of back-isomerisation. Achieving this high level of control has a direct impact on the release of energy on-demand, which affects several applications, such as photochemical actuation^93^ or molecular solar thermal energy storage (MOST).^94^

Azobenzenes offer an efficient platform to showcase the different strategies implemented to this end. Most approaches aim to diminish the double-bond character of the N=N group to lower the barrier of isomerisation of the metastable state. Similarly to the photochemical oxidative process presented in the previous section, electron and hole catalysis have been implemented both with chemical oxidants and electrochemistry to regulate the lifetime of the *Z* form of azobenzenes.^32,86^ Ablated gold,^85,95,96^ zirconium oxide^97^ and silver^98^ nanoparticles were also found to be excellent oxidants for the thermal *Z*–*E* conversion of azobenzenes. Alternatively, the use of Lewis bases and acids^99^ as well as protic acid sensitisers^100^ is well known and reported in the literature. Along this line, by modifying the structure of the azobenzene to accommodate groups bearing acidic protons (*e.g.* NH or OH), it is possible to regulate the rate of thermal back-relaxation by modulating the water content of the solvent mixture, to promote the formation of the corresponding hydrazone tautomers.^101,102^ Similarly, a more general effect on the water concentration on the thermal back isomerisation of photoswitches was discovered in monoarylated indigo derivatives monosubstituted with an NH.^103^ In this particular case, the solvent interactions lead to a 300-fold enhancement of the thermal reaction of the metastable form of the switch.

**Fig. 6:**
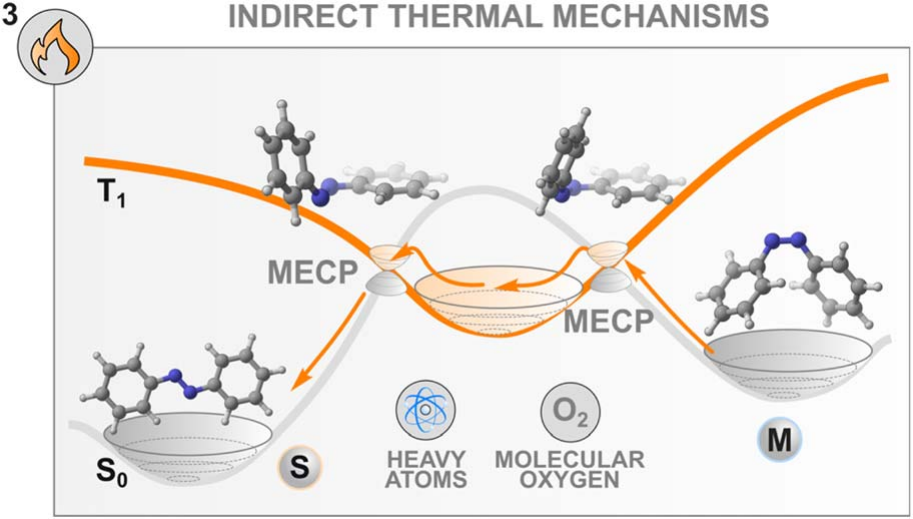
The mechanism of thermal isomerisation of azobenzene involves crossing to the T_1_ state *via* two minimum energy crossing points (MECPs). The presence of heavy atoms and molecular oxygen influence the thermal process.

Herein, however, we would like to underline another peculiarity of the thermal isomerisation of azobenzenes which was overlooked until recently in the literature.^104^ Since the discovery of its isomerism in 1937,^105^ the nature of the pathway that the molecule follows both at the excited and at the ground states were the source of fierce debates. During the thermal event, two mechanistic pathways were classically considered, inversion around or rotation about the central N=N azo bond following a classical adiabatic mechanism on the singlet ground state surface,^106–108^ with results often influenced by the substituents and the level of constrain in the molecule.^109^ Nevertheless, the experimental evidence was not consistently backed up by the computational interpretation of the results. Most strikingly, the entropy of activation that is experimentally obtained does not follow the sign (for the inversion) and the magnitude (for the rotation) of the computed ones, affording the so-called “entropy puzzle”.^110^ An alternative mechanism for the azobenzene thermal isomerisation was first proposed in 2004 in a computational investigation of the excited state decay and thermal isomerisation routes of *Z*-azobenzene on the S_0_, S_1_, and T_1_ states.^47^ According to this study, the thermal reaction proceeds following a multistate process involving both the S_0_ and T_1_ states which cross in a region close to the transition state associated with the rotation mechanism (Fig. 6). Interestingly, this same multistate mechanism was proposed in 1941, where the authors discussed the potential for rotation about an ethylenic double bond to occur in two fashions. This proposal was later rejected for molecules bearing C=C double bonds.^111^

Recently, the thermal population of the lowest triplet state of azobenzenes was probed experimentally.^104^ This multistate mechanism involves two surface crossings involving the S_0_ and T_1_ states and is classified as a type II rotation mechanism (Fig. 6) to differentiate it from the adiabatic one (type I rotation). To describe such a mechanism, Eyring's transition state theory (TST) is no longer valid, therefore non-adiabatic TST needs to be applied. The S_0_ and T_1_ surfaces intersect at two minimum energy crossing points which play a similar role to the transition state in conventional Eyring's TST. In this case, the transmission coefficient depends explicitly on the spin–orbit coupling.^103^ The mechanism seems general for the different azoarenes.^112–114^

These new insights into the mechanism of azobenzene offer a fresh perspective on the thermal control of the stability of the metastable state of a switch. Indeed, control of the thermal back-isomerisation can be exerted by using additives with heavy atoms in the solvent mixture that can modulate the spin–orbit coupling between the surfaces with different multiplicities and facilitate (or hinder) the thermal back-isomerisation. This was demonstrated in a study where 0.25 M tetrabutylammonium iodide in the solution (of 60 μM azobenzene) slightly accelerated the *Z* → *E* isomerisation, especially at elevated temperatures, by increasing the activation entropy.^104^ The acceleration of thermal isomerisation was earlier observed in the presence of palladium and platinum porphyrins^29^ as well as molecular iodine^87^ but these effects were not investigated, making it unsure whether they originate from the heavy atom effect or other interactions such as coordination to the nitrogen lone pairs.

Interestingly, the thermal isomerisation of some switches is apparently dependent on a triplet quencher. BF_2_-coordinated azo complexes show an unusual influence of oxygen on the thermal relaxation of the metastable state, increasing the switching rate more than twenty-fold.^115^ Also the thermal isomerisation promoted by molecular iodine seems to be oxygen-dependent.^87^ The origin and generality of this effect are yet unknown, but it could possibly be leveraged for controlling the thermal back-isomerisation of at least some photoswitches.

These results are the corollary of a scientific story started in 1941 but open more possibilities for the precise control of the thermal isomerisation of molecular switches by multiple surface-crossings. Even though for C=C double bonds the mechanism was ruled out, other switches, especially azobenzenes, do manifest peculiar thermal behaviour in the presence of heavy atoms or triplet quenchers. Recent findings demonstrate that (i) this phenomenon can be exploited to control the back-isomerisation rate without a light stimulus, and that (ii) there are still details in the isomerisation that we have not completely unveiled.

## Conclusions and outlook

A wide range of photoswitches have been developed starting from the mid-20th century, each showing unique nanoscopic changes upon photoisomerisation that, when incorporated into a larger structure, can bring about macroscopic changes in properties ranging from colour and solubility to shape and motion. Owing to the large variety of different photoswitch structures, these molecules have found use in many fields of chemistry, physics, biology, and materials science. To meet the requirements of such diverse application areas, substitution patterns have been designed that tune the essential photoswitching parameters, including the wavelengths the switches absorb, the spectral separation of the two isomers and consequently the photostationary state isomer distributions, quantum yields of isomerisation, and the thermal stability of the metastable isomer. Particular efforts have been made to shift the absorption wavelengths away from the ultraviolet region and even to the red end of the visible spectrum – or beyond, to the near-infrared region. However, it has proven difficult to decouple the absorption profile from other properties: typically, structural changes that shift absorption towards the red end of the visible spectrum also lead to fast thermal back-isomerisation as well as diminished robustness, which is an obstacle to their wide use. Indirect photoisomerisation, based on exciting a proxy system that induces the isomerisation event, provides a way to using longer wavelengths without making changes to the photoswitch core, thus keeping its favourable properties intact. In this perspective, we have explored four different indirect excitation methods that can be applied to photoswitching: (i) two-photon excitation and subsequent singlet energy transfer, (ii) triplet sensitisation, (iii) photon upconversion, and (iv) photoinduced electron transfer. We have also introduced thermal processes that can be utilised to affect the isomerisation of certain photoswitches. For each indirect isomerisation strategy, we have provided theoretical background, state-of-the-art examples, and practical considerations.

Each approach possesses unique prospects for applications. Two-photon excitation of an antenna and subsequent singlet energy transfer can be used to drive the isomerisation in either direction with near-infrared light, but it requires a focused high-power laser beam even with a good absorber. This method enables local isomerisation with extremely high precision, which could be useful for certain applications, but also makes uniform isomerisation in a large sample impossible, narrowing the applicability of this strategy. In addition, the absorber needs to be covalently linked to the photoswitch, which poses additional synthetic strain compared to other strategies that can be exploited by using separate sensitiser molecules with low-power, non-coherent light sources such as LEDs or the sun. Like two-photon excitation, photon upconversion can be leveraged for isomerisation in either direction as the conversion of low-energy photons into high-energy photons is decoupled from the isomerisation events; however, this usually only works if the upconversion system and the photoswitch are physically separated. Otherwise, energy or electron transfer events can take place before upconverted fluorescence, which may be an impediment but also a pathway to eloquent isomerisation mechanisms. The remaining two approaches do not have such a requirement. The function of triplet sensitisation depends on the photoswitch – for instance, stilbenes and diazocines can be isomerised in either direction by choosing suitable sensitisers, while the sensitisation of azoarenes yields mostly the *E* isomer and thus only catalyses the *Z* → *E* isomerisation unless novel strategies such as isomer-specific confinement (the DESC approach) are implemented. The biggest drawback related to working within the triplet manifold is the quenching by molecular oxygen that can pose challenges to many otherwise potential applications, although properly chosen strategies to shield the systems from oxygen or scavenge it may solve this issue. In this sense, isomerisation *via* photoinduced electron transfer is a more robust method. Moreover, thanks to the chain reaction character of indirect photoswitching induced by PET, a quantum yield of 199% may be reached.^32^ This is in striking contrast to the QY values obtained employing triplet sensitisation (up to 28%)^28,32^ and DESC (6.4%).^64^ However, it can only induce isomerisation from the metastable to the stable state (*Z* → *E* in the case of azoarenes and stilbenes) and has, so far, a rather limited scope. The same negatives apply to indirect thermal methods. However, each indirect excitation method can potentially benefit some application areas.

Indirect photoswitching has so far provided intriguing alternative handles for manipulating photoswitches. These efforts have revealed both fundamental (photo)physicochemical properties of different photoswitching families and improved or even introduced new applications for them. As stated above, it is foreseen that the applications that can most benefit from indirect photoswitching would in general meet the following criteria: (i) the absorption band(s) of the molecule are inconvenient in terms of the intended use (*e.g.* biological samples and UV absorption), and (ii) the photoswitch cannot be chemically altered to fine-tune its photoswitching properties without changing the application–relevant properties. As both of these are true for biological applications of many photoswitches,^116^ it is expected that many future applications could be found in biomedicine and biotechnology.

The fundamental side of the field presents at least two clear avenues of research: (i) expanding the scope of indirect photoswitching to new photoswitch families and (ii) systematic studies on structure–property relationships on different sensitiser–photoswitch pairs. Under more applied themes, fearless and unprejudiced probing into new application areas as well as finding ways of circumventing such obstacles as oxygen-sensitivity are required to realise the full potential of indirect photoswitching. The field will undoubtedly benefit from a holistic approach in designing the indirect photoswitching system, where both the thermal (ground state) and photophysical (photoexcited state) properties of the switch and the sensitiser and how they influence each other are considered. This approach requires combined computational, synthetic, and spectroscopic efforts, underlining the complex nature yet vast capabilities and tunability of these systems.

## Data availability

No primary research results, software or code have been included and no new data were generated or analysed as part of this review.

## Author contributions

K. K. and A. P. conceived the idea for the perspective. K. K., J. I., J. J. W., S. C. and N. A. D. wrote the manuscript, with K. K. being primarily responsible for the work. S. C. created the figures. T. L., N. A. D., S. C. and A. P. acquired funding. All authors provided critical feedback and commented on the manuscript.

## Conflicts of interest

There are no conflicts to declare.
